# Advances in Implant Dentistry—A Special Issue of *Dentistry Journal*

**DOI:** 10.3390/dj3040132

**Published:** 2015-11-02

**Authors:** Bernhard Pommer

**Affiliations:** Academy for Oral Implantology, Lazarettgasse 19/DG, 1090 Vienna, Austria; E-Mail: pommer@implantatakademie.at

New knowledge is developing at a rapidly increasing rate in implant dentistry, as in other areas of medicine. The implementation of osseointegrated implants, initiated by Brånemark over 40 years ago, has, without doubt, revolutionized oral medicine. While early research on implant dentistry was primarily concerned with fixture survival, interests are nowadays also focused on simplification of surgical procedures and optimization of single-tooth implant esthetics. Survival rates reported in studies prior to the millennium increased from 93.5% to 97.1%, thereafter (Pjetursson *et al*. 2014 JOMI 29:S308) limiting the potential for further improvement. Progress is instead attempted by application of minimally invasive surgical techniques to reduce patient morbidity and optimize peri-implantitis treatment. This special issue "Advances in Implant Dentistry" aims to review the current state-of-the-art regarding novel surgical and prosthodontic techniques and may, at times, challenge established dogmas, *i.e.*, opinions based more on belief than on evidence-based implant dentistry.

Topics might include but are not limited to:

- template-guided implant placement;
- minimally invasive techniques;
- short lengths and reduced implant diameter;
- novel bone grafting techniques;
- medically compromised patients;
- peri-implantitis treatment;
- immediate placement and restoration;
- transition from a failing dentition;
- CAD/CAM prosthetics;
- optical intraoral impressions.

